# Biochemical characterization of protein quality control mechanisms during disease progression in the C22 mouse model of CMT1A

**DOI:** 10.1042/AN20130024

**Published:** 2013-12-03

**Authors:** Vinita G. Chittoor, Lee Sooyeon, Sunitha Rangaraju, Jessica R. Nicks, Jordan T. Schmidt, Irina Madorsky, Diana C. Narvaez, Lucia Notterpek

**Affiliations:** *Departments of Neuroscience and Neurology, College of Medicine, McKnight Brain Institute, University of Florida, Gainesville, FL, U.S.A.

**Keywords:** autophagy, chaperone, ubiquitin, myelin, protein aggregation, Schwann cell., AMC, amino-methyl coumarin, CathD, Cathepsin D, CMT1A, Charcot–Marie–Tooth disease type 1A, di-8-ANEPPS, 4-[2-(6-dibutylamino)-2-naphthalenyl)ethenyl]-1-(3-sulfopropyl) hydroxide, Egr2, early growth response 2, endoH, endoglycosidase H, ER, endoplasmic reticulum, HRP, horseradish peroxidase, HSF1, heat-shock factor 1, Hsp, heat-shock protein, IgG, immunoglobulin, LAMP1, lysosomal membrane-associated protein 1, LC3, light chain 3, MCP-1, monocyte chemoattractant protein 1, MS, multiple sclerosis, Oct6, octamer-binding transcription factor 6, PMP22, peripheral myelin protein 22, PNGaseF, N-glycosidase F, pUb, polyubiquitinated, TFEB, transcription factor EB, UPS, ubiquitin–proteasome system, Wt, wild-type

## Abstract

Charcot–Marie–Tooth disease type 1A (CMT1A) is a hereditary demyelinating neuropathy linked with duplication of the peripheral myelin protein 22 (*PMP22*) gene. Transgenic C22 mice, a model of CMT1A, display many features of the human disease, including slowed nerve conduction velocity and demyelination of peripheral nerves. How overproduction of PMP22 leads to compromised myelin and axonal pathology is not fully understood, but likely involves subcellular alterations in protein homoeostatic mechanisms within affected Schwann cells. The subcellular response to abnormally localized PMP22 includes the recruitment of the ubiquitin–proteasome system (UPS), autophagosomes and heat-shock proteins (HSPs). Here we assessed biochemical markers of these protein homoeostatic pathways in nerves from PMP22-overexpressing neuropathic mice between the ages of 2 and 12 months to ascertain their potential contribution to disease progression. In nerves of 3-week-old mice, using endoglycosidases and Western blotting, we found altered processing of the exogenous human PMP22, an abnormality that becomes more prevalent with age. Along with the ongoing accrual of misfolded PMP22, the activity of the proteasome becomes compromised and proteins required for autophagy induction and lysosome biogenesis are up-regulated. Moreover, cytosolic chaperones are consistently elevated in nerves from neuropathic mice, with the most prominent change in HSP70. The gradual alterations in protein homoeostatic response are accompanied by Schwann cell de-differentiation and macrophage infiltration. Together, these results show that while subcellular protein quality control mechanisms respond appropriately to the presence of the overproduced PMP22, with aging they are unable to prevent the accrual of misfolded proteins.

## Abstract 2

In peripheral nerves of neuropathic C22 mice the frequency of cytosolic PMP22 aggregates increases with age, which elicits a response from protein quality control mechanisms. The combined effects of aging and neuropathic genotype exacerbate disease progression leading to nerve defects.

## INTRODUCTION

Charcot–Marie–Tooth disease type 1A (CMT1A) hereditary demyelinating peripheral neuropathies are linked with duplication or point mutations in the peripheral myelin protein 22 (*PMP22*) gene (Young and Suter, 2001). While PMP22 is known to be expressed in a number of different cell types, nerve transplantation studies established that the neuropathy initiates within the Schwann cells (Aguayo et al., 1977). One transgenic rodent model of CMT1A, termed C22, has integrated seven copies of the human *PMP22* gene and expresses ˜1.7-fold more human PMP22 mRNA than the endogenous mouse transcript (Huxley et al., 1996). By 6 months of age, affected mice develop prominent motor impairments, nerve demyelination and muscle atrophy (Norreel et al., 2003; Fortun et al., 2006; Szigeti and Lupski, 2009). Examination of nerves from 6-month-old C22 mice revealed Schwann cells with abnormal cytosolic PMP22 aggregates that were reactive for ubiquitin and were surrounded by autophagosomes and lysosomes (Fortun et al., 2006). The presence of such abnormal protein aggregates was associated with an impairment of proteasome activity, which is a commonality among PMP22 point mutation and gene duplication CMT1A paradigms (Fortun et al., 2005, 2006). Intracellular retention, including cytosolic accumulation of PMP22 has been observed in nerves from symptomatic CMT1A patients (Nishimura et al., 1996; Hanemann et al., 2000) indicating that age-associated changes in subcellular protein homoeostatic mechanisms likely contribute to the pathogenesis of the disease.

In protein misfolding disorders such as CMT1A, cells activate subcellular defense mechanisms which either support protein refolding or target them for degradation (Sherman and Goldberg, 2001; Williams et al., 2006). Protein quality control pathways that help to maintain cellular homoeostasis include the ubiquitin–proteasome system (UPS), the chaperones, and macroautophagy. The UPS is a particularly important mechanism in PMP22 neuropathies, as the proteasome is responsible for the degradation of newly synthesized, short-lived PMP22 (Pareek et al., 1997; Notterpek et al., 1999). Macroautophagy (hereafter referred to as autophagy) is also critical in PMP22-linked neuropathies as autophagosomes accumulate near protein aggregates within neuropathic Schwann cells and under permissive conditions, activating autophagy clears the misfolded PMP22 (Fortun et al., 2003, 2006, 2007). The third defense mechanism involves molecular chaperones that can prevent protein aggregation by assisting folding (Young et al., 2004) or degradation (Vashist et al., 2010).

In humans a characteristic feature of CMT1A is the progressive nature of the disease which typically surfaces in the second decade of life (Jani-Acsadi et al., 2008; Szigeti and Lupski, 2009). While disease progression is a critical aspect of the neuropathies, there have been a limited number of studies examining affected nerves at different stages of life-span. In view of disease progression, it is important to consider normal aging-associated degenerative events in myelinated nerves, which include morphological and biochemical changes such as demyelination, widening of the nodes of Ranvier and accumulation of collagen and lipid droplets (Ceballos et al., 1999; Rangaraju et al., 2009; Opalach et al., 2010). Dietary modulation, including life-long calorie restriction or extended intermittent fasting are two approaches that slow aging-associated degenerative events in myelinated peripheral nerves, and both of these interventions influence subcellular protein homeostatic mechanisms (Lee and Notterpek, 2013). Therefore, changes in degradative and chaperone mechanisms with age likely impact the progression of hereditary nerve disorders, particularly where protein misfolding is involved such as in PMP22-linked neuropathies.

While the three mentioned protein homoeostatic mechanisms have been associated with pathobiology of PMP22-linked neuropathies, their potential contribution to disease progression has not been examined in detail. In the current study we examined sciatic nerves from age-matched wild type (Wt) and C22 mice between the ages of postnatal day 21 and 12-months, an age-span that encompasses pronounced clinical, electrophysiological and morphological deficits (Verhamme et al., 2011). Our biochemical and immunohistological studies reveal an age-associated accumulation of the overproduced PMP22, despite evidence for activation of protein homoeostatic mechanisms.

## MATERIALS AND METHODS

### Mouse colonies

C57Bl/6J wild-type (Wt) and PMP22 overexpressor (C22) (Huxley et al., 1996) mouse colonies were housed under SPF conditions at the McKnight Brain Institute animal facility. The use of animals for these studies was approved by University of Florida Institutional Animal Care and Use Committee (IACUC). Genomic DNA was isolated from tail biopsies and litters were genotyped by PCR (Huxley et al., 1996). Sciatic nerves harvested at the indicated time points from male and female mice were pooled (*n*=3–6 animals per age group). We used the 12-month time point as the oldest group, as by this age neuropathic mice have deficits that can interfere with normal grooming behavior.

### Primary antibodies

To detect PMP22 in nerve sections, a 1:1 mixture of two rabbit polyclonal antibodies, developed against a peptide corresponding to the second extracellular loop of the human or the rat PMP22 was used (Pareek et al., 1997). These antibodies have been shown to recognize the mouse PMP22 as the amino acid sequence between rat and mouse shares 94% identity, while the identity is only 81% between the mouse and human sequences (Fortun et al., 2003). All other primary antibodies used in this study are listed in Table 1.

**Table 1:** Primary antibodies used in the present study WB, Western blot; IS, immunostaining; n/a, non-applicable.

Species	Antigen	Source and catalog number	Dilution WB	IS
Mouse	Tubulin	Sigma; T6199	1:2000	n/a
Rabbit	Ubiquitin	Dako; Z0458	1:1000	n/a
Rat	LAMP1	DSHB, University of Iowa	1:200	1:100
Rabbit	Cathepsin-D	Cortex Biochem; CP3090	1:1000	n/a
Mouse	GAPDH	Encor Biotechnology Inc; MCA-1D4	1:10000	n/a
Rabbit	Atg7	Gift from Dr William Dunn Jr, UF	1:500	n/a
Rabbit	LC3	Cell Signaling Technology; 2775	1:1000	n/a
Rabbit	p62	Enzo Life Sciences; PW9860	1:2000	n/a
Rabbit	TFEB	Abcam; ab113372	n/a	1:400
Rabbit	Calnexin	Stressgen; SPA-860	1:1000	n/a
Rabbit	Calreticulin	Stressgen; SPA-600	1:1000	n/a
Goat	HSP27	Santa Cruz Biotechnology, Inc.; sc-1049	1:1000	n/a
Rabbit	αB-crystallin	Stressgen; SPA-223	1:4000	n/a
Rabbit	HSF1	Stressgen; SPA-901	1:1000	n/a
Rabbit	HSP104	Stressgen; SPA-1040	1:100	n/a
Rabbit	HSP90	Cell Signaling Technology; E289	1:1000	n/a
Rabbit	HSP70	Stressgen; SPA-812	1:3000	n/a
Rabbit	HSP40	Stressgen; SPA-400	1:2000	n/a
Mouse	Hypophosphorylated (hp)- NFH	Covance; SMI-32P	1:1000	n/a
Rabbit	Spectrin	Encor Biotechnology Inc.; RPCA-aII-Spec	1:1000	n/a
Rabbit	GAP43	Encor Biotechnology Inc.; RPCA-GAP43	1:1000	n/a
Rabbit	p75	Chemicon; AB1554	1:2000	n/a
Mouse	pHH3 (Ser10)	Millipore; 05-598	1:500	n/a
Rabbit	Oct6	Abcam; ab5969	1:1000	n/a
Rabbit	Egr2	Santa Cruz Biotechnology, Inc; sc-20690	1:200	n/a
Rat	CD11b	Serotec; MCA711	1:1000	1:500
Mouse	Albumin	Abcam; ab19194	1:250	n/a

### Immunohistochemical studies on nerve sections

Sciatic nerves from genotyped 2- and 12-month-old Wt and C22 mice were processed for immunostaining as described (Fortun et al., 2006). Bound primary antibodies were detected with Alexa Fluor 594-conjugated goat anti-rabbit and Alexa Fluor 488-conjugated goat anti-rat IgG, or anti-mouse IgG/IgM (Molecular Probes). Samples without primary antibodies were processed in parallel as negative controls. Coverslips were mounted using the Prolong Antifade kit (Molecular Probes). Images were acquired with a SPOT digital camera (Diagnostic Instrumentals) attached to a Nikon Eclipse E800 or an Olympus DSU spinning disc confocal (Tokyo, Japan) microscope, using the same exposure settings. Images were processed using Photoshop 5.5 (Adobe Systems). To detect the levels of lipofuscin-like substrates within the nerves, the lipophilic dye 4-[2-(6-dibutylamino)-2-naphthalenyl)ethenyl]-1-(3-sulfopropyl) hydroxide (di-8-ANEPPS) was used (Grune et al., 2004; Opalach et al., 2010). For CD11b staining, samples were processed as detailed previously (Misko et al., 2002). To stain for endogenous nerve immunoglobulins (IgG and IgM), primary antibody incubation was omitted and permeabilized sections were directly incubated with Alexa Fluor-conjugated secondary antibodies against mouse IgG and IgM.

### Quantification of immunohistochemical data

In longitudinal sections (5 μm thickness) of sciatic nerves from Wt and C22 mice the number of PMP22-containing protein aggregates were counted in eight random visual fields (0.1 mm^2^) (Fortun et al., 2003, 2006). The nuclei (stained with Hoechst dye) of non-epineurial and non-endoneurial cells were counted in eight random visual fields (0.1 mm^2^) per condition (*n*=3 mice). Total transcription factor EB (TFEB) fluorescence was calculated from six random fields (*n*=3 mice per group), using ImageJ software, with the Correlated Total Cell Fluorescence method. CD11b-positive cells were counted in nine random microscopic fields (0.1 mm^2^) from at least three different nerve sections (*n*=3 mice per condition).

### Western blot analyses

Frozen sciatic nerves from Wt and C22 mice (*n*=3–6 animals per group, per experiment) were processed for Western blotting as described (Rangaraju et al., 2009). Primary antibodies were detected with anti-rat or anti-goat (Sigma–Aldrich) horseradish peroxidase (HRP)-linked secondary antibodies. To detect the levels of endogenous mouse IgGs, incubation with the primary antibodies was omitted and the membranes were directly probed with HRP-linked anti-mouse secondary antibodies. Bound antibodies were visualized using an enhanced chemiluminescence detection kit (PerkinElmer Life Sciences). Films were digitally imaged using a GS-710 densitometer (Bio-Rad Laboratories) and were formatted for printing, using Adobe Photoshop 5.5. The processing of PMP22 was assessed by endoglycosidase H (endoH) or N-glycosidase F (PNGaseF) enzyme (New England Biolabs) digestions, as described (Pareek et al., 1997). All biochemical experiments were repeated at least three times using protein lysates from independent mouse nerve samples. Densitometric analyses of Western blots were done using ImageJ software. Integrated densitometric values of proteins were normalized to their respective loading controls.

### Measurements of 20S proteasome activity

The chymotrypsin-like activity of the 20S proteasome was measured by changes in the fluorescence of amino-methyl coumarin (AMC)-conjugated to the chymotrypsin peptide substrate LLVY (Chemicon) (Fortun et al., 2005). Freshly collected nerves from 1–2 month-old and 12–13-month-old Wt and C22 mice were homogenized and the degradation of LLVY–AMC within the lysates was measured by cleavage of AMC from LLVY. To confirm the specificity of the fluorescence signal obtained in the assay, in a subset of samples, the proteasome inhibitor lactacystin (10 μM) was added. The chymotrypsin-like activity of the 20S proteasome was normalized to μg of protein in each sample (*n*=6–10 mice/condition) and plotted with respect to the positive control (provided in the kit).

### Statistical analyses

For all experiments, mean±S.E.M. was calculated and significance determined by performing unpaired two-tailed Student's *t* tests, using GraphPad Prism software. *P* values <0.05 (*), <0.01 (**) and <0.001 (***) were considered significant.

## RESULTS

### Age-associated increase in PMP22 aggregation and proteasome malfunction

**Figure 1:**
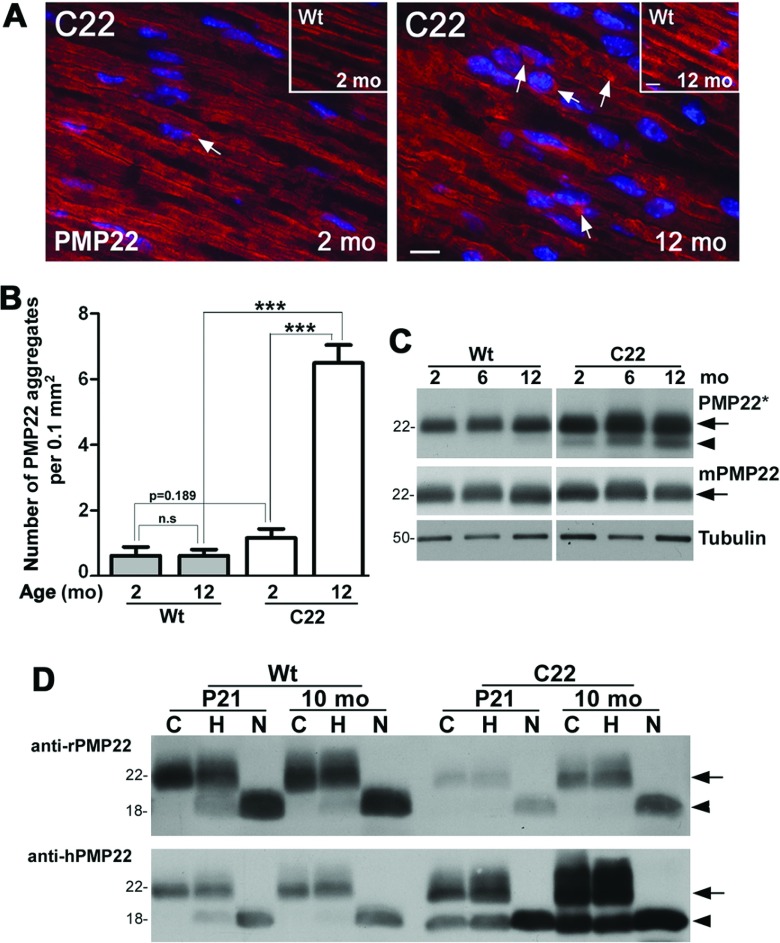
Accrual of PMP22 aggregates in nerves of neuropathic mice (**A**) Sciatic nerve sections from 2- and 12-month-old Wt (insets) and C22 mice were immunolabeled with anti-PMP22 antibodies (red) and Hoechst dye (blue). Intracellular PMP22-reactive structures are marked by arrows. Scale bars, 10 μm. (**B**) The number of PMP22-reactive aggregates was counted in eight random fields (0.1 mm^2^, per field area) of nerve sections. Unpaired Student's *t* test; ***P<0.001; n.s., not significant; mean±S.E.M.; *n*=3–4 mice per group. (**C**) Sciatic nerve lysates (4 μg/lane) from 2-, 6- and 12-month-old Wt and C22 mice (*n*=3–4 mice per group) were blotted with either an antibody mixture that recognizes both mouse and human PMP22 (PMP22*) or only the mouse protein (mPMP22). Tubulin serves as a loading control. (**D**) To assess the post-translational processing of PMP22 at steady-state, total nerve lysates (15 μg/reaction) from 21-day-old (P21) and 10-month-old Wt and C22 mice (*n*=3–4) were incubated without enzyme (C), with Endoglycosidase H (endoH, H) or N-Glycosidase F (PNGaseF, N) and blotted with antibodies that recognize either the mouse (anti-rPMP22) or the human (anti-hPMP22) protein. The arrows indicate the endoH-resistant (˜22 kDa) and the arrowheads mark the native core (˜18 kDa) PMP22 protein (**C** and **D**). Molecular mass is shown in kDa (**C** and **D**).

Previously, in nerves of 6-month-old C22 mice we detected PMP22 in detergent-insoluble aggregates which fulfilled the criteria for aggresomes (Fortun et al., 2003, 2006). To determine the incidence of such structures with neuropathy progression, we immunostained nerve sections from 2- and 12-month-old Wt and C22 mice with a mixture of anti-PMP22 antibodies that recognizes both the mouse and human protein (Figure 1A). Consistent with previous reports (Notterpek et al., 1997), at 2- and 12-months of age PMP22 is distributed along myelinated axons in nerves of Wt mice (Figure 1A, insets). In comparison, in samples from young C22 mice PMP22-reactive aggregates are seen near Schwann cell nuclei and such structures become more frequent by 1 year of age (Figure 1A, arrows). Quantification of PMP22 aggregates in eight random visual fields of nerve sections from each age and genotype (*n*=3 mice per group) reveals a ˜6-fold increase between 2 and 12-months in the neuropathic samples, while aggregates are rare in normal nerves (Figure 1B). Thus by 1 year of age, the nerves of C22 mice contain significantly more PMP22 aggregates than at 2 months, or Wt counterparts at the same age.

The linkage of elevated PMP22 expression to demyelination (Perea et al., 2001) and the age-associated increase in the prevalence of PMP22 aggregates in affected samples (Figure 1B), prompted us to examine the steady-state levels of PMP22 in nerve lysates from 2-, 6- and 12-month-old Wt and C22 mice (Figure 1C). Using the same anti-rat and anti-human PMP22 antibody mixture (PMP22*) as for the immunostaining in Figure 1(A), we detect a gradual increase in the steady-state levels of PMP22 in affected nerves, including a faster migrating ˜18 kDa form (Figure 1C, arrowhead). Since this reactivity is a combination of the endogenous mouse protein and the transgenic human PMP22, we analyzed the same set of samples with the anti-rat PMP22 antibody alone, which has higher affinity for the mouse protein (mPMP22) as compared with the human. The antigenic peptide of the anti-rat PMP22 antibody shares 94% identity with the mouse peptide, while the identity is only 81% between the rat or mouse, and human sequences. The anti-rat PMP22 antibody reveals a rather uniform expression of PMP22 across the studied samples, including Wt and C22 (Figure 1C). Therefore the increase in the levels of PMP22 in 12-month-old C22 nerves detected with the antibody mixture may be attributed to inefficiency in the processing of the overproduced human protein, which in turn could lead to the build-up of intracellular aggregates.

To further examine the processing of the overproduced PMP22 we took advantage of the preferential reactivity of our anti-PMP22 antibodies to the human or the mouse proteins, following treatment of the samples with endoglycosidases (Pareek et al., 1997). Whole nerve lysates from postnatal day 21 (P21) and 10-month-old Wt and C22 mice were incubated in control buffer (C), endoglycosidase H (H) or N-glycosidase F (N) and blotted with anti-rat or anti-human PMP22 antibodies (Figure 1D). As shown previously (Pareek et al., 1997), at steady-state, in nerves of Wt mice the majority of PMP22 is resistant to endoH (Figure 1D, arrows), which indicates processing of the protein past the medial-Golgi compartment. N-glycosidase F removes all carbohydrate modification from PMP22, revealing the 18 kDa core peptide (Figure 1D, arrowheads). As shown in Figure 1(D) (upper panel), the anti-rat PMP22 antibody is more sensitive in detecting the Wt mouse protein, as compared with the anti-hPMP22 antibody (compare first six lanes between upper and lower blots). On the other hand, the anti-rPMP22 antibody is weakly reactive against the C22 samples, but the processing of the recognized, likely endogenous mouse PMP22, is similar to that in Wt. Significantly, the anti-hPMP22 antibody shows a preferential reactivity against the neuropathic samples and reveals the expression of an ˜18 kDa form, which is also seen in Figure 1(C) when the antibody mixture is used (PMP22*). In samples from 10-month-old C22 mice, the endoH resistant ˜22 kDa (Figures 1C and 1D, arrows) and ˜18 kDa forms of PMP22 comprise about 50% of the total protein, which is a distinct pattern from that seen in Wt. Therefore it is likely that this incompletely processed ˜18 kDa form of PMP22 represents the misfolded, aggregated protein present at elevated levels in older neuropathic samples.

**Figure 2:**
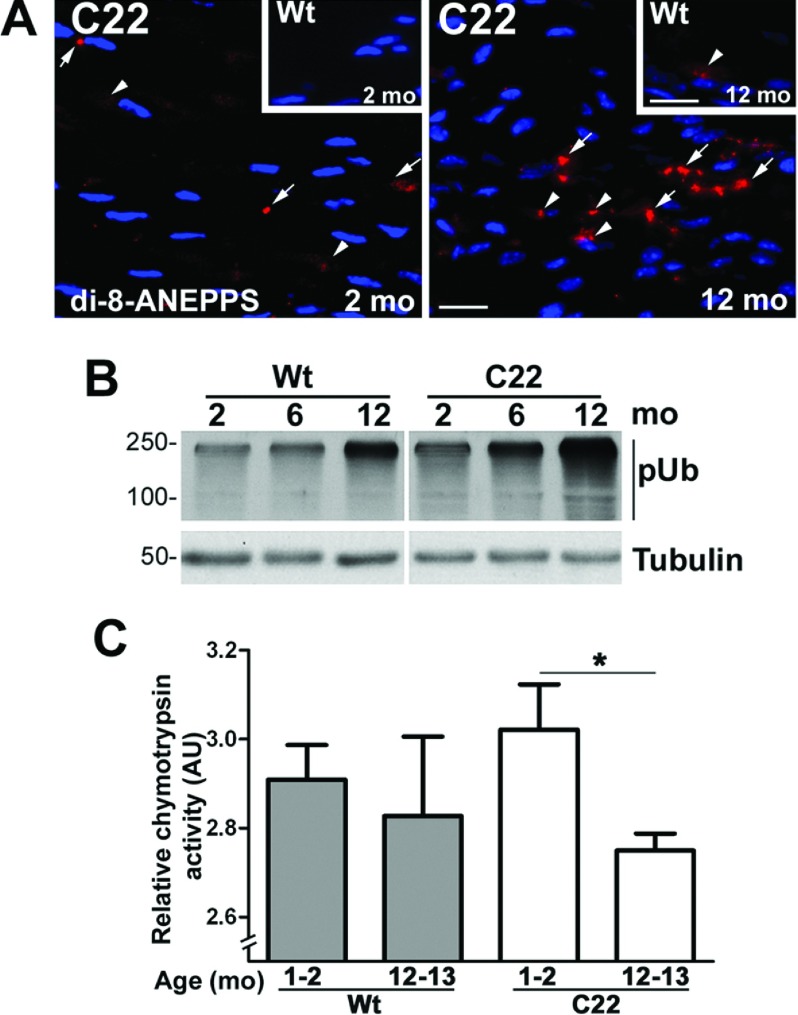
Progressive decline in proteasome function in affected nerves (**A**) Sciatic nerve sections from 2- and 12-month-old Wt (insets) and C22 mice were stained with di-8-ANEPPS dye (red). Clumps of di-8-ANEPPS-positive adducts are marked with arrows, and arrowheads indicate smaller or scattered adducts. Nuclei are labeled with Hoechst dye (blue). Scale bars, 20 μm. (**B**) The levels of slow migrating poly-ubiquitinated (pUb) substrates were analyzed in sciatic nerve lysates (10 μg/lane) from 2-, 6- and 12-month-old Wt and C22 mice (*n*=3–4 mice per group). Tubulin is shown as a protein loading control. Molecular mass is shown in kDa. (**C**) The 20S chymotrypsin-like activity of the proteasome was assayed in sciatic nerve lysates from 1–2- and 12–13-month-old Wt and C22 mice (*n*=8–9 mice per group). The activity in each sample relative to the positive control is plotted on the *y*-axis. Unpaired Student's *t* test; *P<0.05; mean±S.E.M.; AU, arbitrary units.

The accumulation of misfolded and damaged proteins with aging is believed to be a major contributor to the progression of late-onset neurodegenerative disorders and is known to occur even in normal aged peripheral nerves (Terman and Brunk, 2004; Opalach et al., 2010). To monitor the general accrual of undegraded lysosomal substrates during the age-span of our mice, we reacted nerve sections with the lipophilic di-8-ANEPPS dye (Figure 2A) (Grune et al., 2004). Whereas lipofuscin-like staining is rarely detected in nerves from Wt animals (Figure 2A, insets), prominent di-8-ANEPPS reactivity is evident in neuropathic samples, at both ages. The discovery of di-8-ANEPPS-positive structures in nerves from 2-month-old C22 mice agrees with morphological signs of early axonal degeneration at this age (Verhamme et al., 2011). The prominence of lipofuscin adducts in samples from 12-month-old neuropathic mice is likely the result of age-associated accrual of misfolded and oxidized residues, including the aggregated PMP22 (Figure 1).

One mechanism by which undegraded damaged molecules may alter protein homoeostasis within cells is by inhibiting the activity of the proteasome. To assess potential changes in proteasomal activity with neuropathy progression in a general manner, we determined the steady-state levels of undegraded polyubiquitinated (pUb) substrates in Wt and affected nerves using Western blotting (Figure 2B). In samples from normal animals, there is a notable increase in pUb-tagged proteins between the ages of 2 and 12 months, a finding that agrees with our previous report in normal rats (Rangaraju et al., 2009). However, this trend is more pronounced in nerve samples from C22 mice and agrees with the increase in misfolded PMP22 with disease progression (Figure 1). As PMP22 is a substrate for proteasomal degradation, next we examined the degradative capacity of the 20S proteasome using a commercial enzyme assay kit (Figure 2C). The chymotrypsin-like activity of the 20S proteasome corresponds to the rate-limiting step in substrate degradation and can be measured by the cleavage of the LLVY peptide to release the fluorescent moiety, AMC (Fortun et al., 2005). Although there is a slight decrease in proteasomal activity of nerves from Wt mice at 12–13 months of age, it does not reach significance compared with 2-months (Figure 2C). In comparison, proteasomal activity declines by ˜30% (Figure 2C) in 12–13 month-old C22 animals as compared with 1–2 month-old. Together these results indicate that gradual accumulation of misfolded PMP22 and other undegraded pUb proteasome substrates impair the normal functioning of the proteasome, as detected by a chymotrypsin peptide reporter.

### Alterations in protein degradative mechanisms during progression of neuropathy

**Figure 3:**
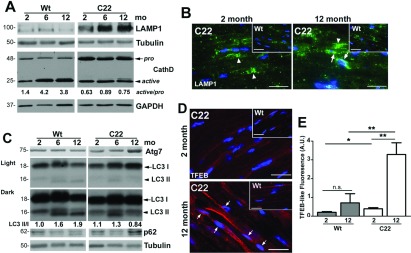
Elevated levels of autophagy–lysosomal proteins in nerves of C22 mice (**A**) The steady-state levels of lysosomal proteins, LAMP1 and cathepsin D (CathD) in total protein lysates of sciatic nerves (10 μg/lane) from 2-, 6- and 12-month-old Wt and C22 mice (*n*=3–4 per group) were analyzed by Western blotting. Arrow points to the 48 kDa pro-cathepsin D (CathD) and arrowhead marks the 28 kDa active CathD. Values of active/pro-CathD ratios are shown below the blot. (**B**) Localization of LAMP1 (green) was analyzed in sciatic nerves from 2- and 12-month-old Wt (insets) and C22 mice (*n*=3 mice per group). Arrowheads point to the paranodal localization of LAMP1, while arrows indicate perinuclear LAMP1-positive structures. (**C**) The same lysates (10 μg/lane) as in (**A**) were analyzed for autophagy markers Atg7, LC3 and p62. LC3 I and LC3 II are marked by an arrow and arrowhead, respectively. Light (upper panel) and dark (lower panel) exposures for this protein are shown. Values of LC3 II/I ratios are shown below the blot. Tubulin (**A** and **C**) and GAPDH (glyceraldehyde-3-phosphate dehydrogenase) (**A**) act as the loading controls. Molecular mass is shown on the left, in kDa (**A** and **C**). (**D**) Single-plane confocal images of sciatic nerve sections from 2- and 12-month-old Wt (insets) and C22 mice, stained for TFEB (red), are shown. Arrows point to TFEB-reactive nuclei. Nuclei are labeled with Hoechst dye (blue) (**B** and **D**). Scale bars, 20 μm (**B** and **D**). (**E**) TFEB fluorescence was quantified in six random visual fields and graphed (*n*=3 mice per group). Unpaired Student's *t* test; *P<0.05; **P<0.01; n.s., non-significant; mean±S.E.M.; A.U., arbitrary units.

The autophagy–lysosomal pathway has the capacity to remove non-functional, damaged proteins, including aggregates of PMP22 (Fortun et al., 2007), yet as shown above, misfolded PMP22 accumulates in nerves of C22 mice. To determine if there is an age-related change in the response of the autophagy–lysosomal pathway to increased PMP22 aggregation (Figure 1) and reduced proteasome activity (Figure 2), we analyzed whole nerve lysates for lysosomal membrane-associated protein 1 (LAMP1) and Cathepsin D (CathD), a lysosomal protease (Cataldo et al., 1995) (Figure 3A). The level of LAMP1 remains relatively steady during the studied life-span in Wt nerves, as described previously (Notterpek et al., 1997). In comparison, nerves of C22 mice contain higher levels of LAMP1 already at 2 months, which further increase between 2 and 6 months and remain elevated at 1 year. Similarly, the expression of total CathD (pro+active) is consistently higher in neuropathic nerves, as compared with Wt. Nonetheless, the ratio of active CathD (28 kDa, arrowhead) to pro-CathD (48 kDa, arrow) isoforms increases in normal aging (1.4 to 3.8) while it remains relatively steady in C22 (0.63 to 0.75). This trend in the ratios of the steady-state CathD isoforms might be the result of impaired lysosomal proteolysis or defective CathD trafficking in nerves of C22 mice. To investigate the localization of lysosomes, we immunostained frozen sections of sciatic nerves from 2- and 12-month-old mice with anti-LAMP1 antibodies (Figure 3B). In nerves of normal mice, LAMP1-positive structures are mostly confined to paranodal regions (Figure 3B, arrowheads), as seen before (Notterpek et al., 1997). In comparison, samples from C22 mice have an abundance of LAMP1-reactive lysosomes not only near paranodes (arrowheads), but also in the vicinity of Schwann cell nuclei (Figure 3B, arrows).

Undegraded, aggregated proteasome substrates are delivered for lysosomal degradation by macroautophagy. Therefore next we examined the steady-state levels of autophagic proteins within the same nerve lysates (Figure 3C). Autophagy-related gene 7 (Atg7), microtubule-associated protein light chain 3 (LC3) and p62 are considered markers of autophagy induction, autophagosomes, and autophagic cargo, respectively (Klionsky et al., 2012). The levels of Atg7, an enzyme essential for the conjugation events that lead to autophagosome formation (Ohsumi, 2001) are elevated at 12-months in samples from affected animals, but not in age-matched Wt (Figure 3C). LC3 I is an autophagy protein which upon lipidation is converted into LC3 II and is incorporated into autophagosomes. Therefore a higher LC3 II/LC3 I ratio is indicative of increased autophagic activity, decreased clearance or both (Klionsky et al., 2012). Although the ratio of LC3 II/LC3 I goes up nearly 2-fold with age in nerves from Wt animals (1.0 to 1.9), this ratio is fairly steady in samples from neuropathic mice (1.1 to 0.84). In comparison, the levels of p62, a linker protein between pUb protein aggregates and autophagy (Bjorkoy et al., 2009), are elevated in nerves of 12-month-old C22 mice, likely as a reflection of undegraded substrates (Figure 2). As described in the Materials and methods section, each of the lanes on the Western blots represent nerves pooled from three to five animals and the data shown is representative of independent batches of nerves.

Since the autophagy–lysosomal proteins examined in Figures 3(A)–3(C) are target genes of TFEB (Settembre and Ballabio, 2011), we immunostained nerve sections with a polyclonal anti-TFEB antibody (Figure 3D). As indicated on the representative micrographs, there is a general up-regulation of TFEB-like immunoreactivity in affected nerves, with some immunoreactivity localized to the nuclei of Schwann cells (Figure 3D, arrows). Since this antibody does not yield reliable results on Western blots, we quantified the TFEB-like immunoreactivity from independent nerve sections using 40× images obtained with equal acquisition parameters and ImageJ software (NIH). As shown on the histogram, we detect a nearly 7-fold increase in TFEB-like immunofluorescence in nerves of 12-month-old C22 mice as compared with Wt or 2-month-old C22 mice (Figure 3E). Together, the prominent age-associated increase in LAMP1 and p62, as well as Atg7 and TFEB, in nerve samples from 1 year of C22 mice suggests a growing demand on the autophagy–lysosomal pathway with disease progression. Nonetheless, the lack of concomitant increase in active CathD or in the ratio of LC3 II/LC3 I implies that although autophagy is activated, it may be unable to degrade the cargo, which ultimately leads to the accumulation of protein aggregates (Figures 1 and 2).

### Protein chaperones in neuropathic nerves

**Figure 4:**
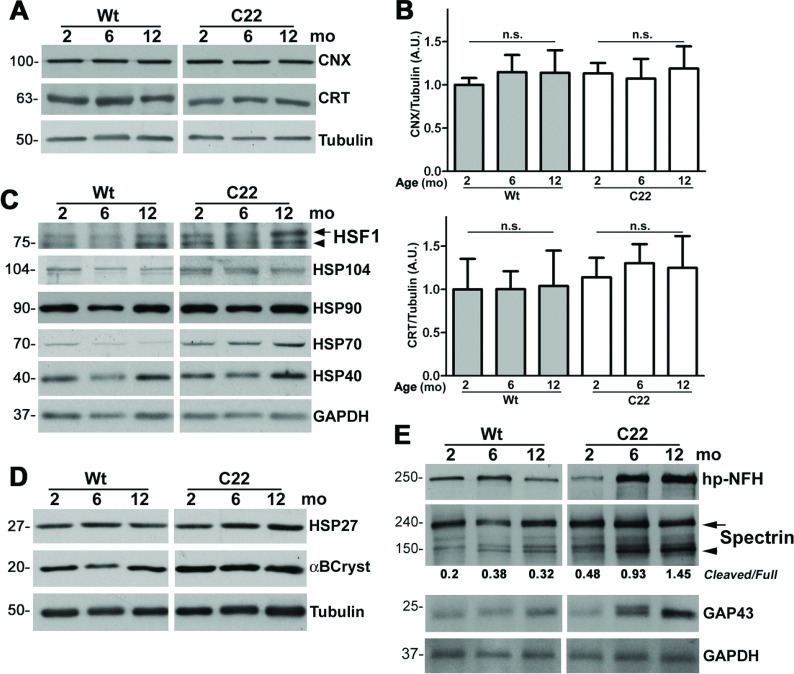
Neuropathy-associated changes in chaperones and axonal proteins in nerves of affected mice (**A**) The levels of ER chaperones, calnexin (CNX) and calreticulin (CRT), were determined in whole nerve lysates (12 μg/lane) from Wt and C22 mice (*n*=3–4 per group). (B) Results of semi-quantitative densitometric analyses (*n*=4 independent experiments) for normalized CNX (top panel) and CRT (bottom panel), after correction for tubulin, are shown. Unpaired Student's *t* test; n.s., non-significant; mean±S.E.M.; A.U., arbitrary units. (**C**) The steady-state levels of the heat-shock factor 1 (HSF1) and the major cytosolic heat-shock proteins HSP104, HSP90, HSP70, HSP40 were analyzed by Western blotting (10 μg/lane). Arrow and arrowhead point to the phosphorylated and non-phosphorylated forms of HSF1, respectively. The same nerve lysates were analyzed for the levels of small heat shock proteins, HSP27 and αB-crystallin (αBCryst) (12 μg/lane) (**D**) and for hypophosphorylated (hp) NFH (hp-NFH, SMI-32 antibody), spectrin cleavage and GAP43 (15 μg/lane) (**E**). Arrow indicates the full-length spectrin and the arrowhead marks the cleaved form. Values of cleaved/full-length spectrin ratios are shown below the blot. Tubulin (**A** and **D**) and GAPDH (glyceraldehyde-3-phosphate dehydrogenase) (**C** and **E**) are shown as protein loading controls. Molecular mass is shown on the left, in kDa (**A**, **C**, **D** and **E**).

Along with protein degradation mechanisms, cytosolic chaperones serve a role in preventing and eliminating misfolded proteins. Previously we have shown that in cell culture transfection paradigms, and in nerves of neuropathic mice, PMP22 aggregates associate with protein chaperones, including αB-crystallin and heat-shock protein 70 (Hsp70) (Fortun et al., 2007). Altered expression of chaperones was also detected in myelinated nerves of 38-month-old aged rats, which had pronounced myelin abnormalities (Rangaraju et al., 2009). Thus we examined the levels of several protein chaperones and the transcription factor heat-shock factor 1 (HSF1) within whole nerve lysates from Wt and C22 mice during the studied age-span (Figures 4A–4D). Since PMP22 has been shown to interact with calnexin (Dickson et al., 2002), first we examined the levels of the endoplasmic reticulum (ER) chaperones calnexin and calreticulin (Figure 4A). Similar to previous findings in the TrJ mouse model of PMP22 neuropathies (Dickson et al., 2002), overproduction of PMP22 in the C22 mice does not elicit an ER stress response. To substantiate this finding, we analyzed the levels of calnexin and calreticulin in three independent sets of samples, after correction for tubulin. As shown in the histograms (Figure 4B), the steady-state levels of calnexin and calreticulin are not significantly different across the studied samples.

Within the same nerve lysates we also investigated the steady-state expression of HSF1, the transcriptional regulator of the heat-shock response. Phosphorylation of HSF1 indicates activation of the chaperone pathway, which involves the translocation of the protein into the nucleus (Westerheide and Morimoto, 2005). As seen previously in nerve samples from 38-month-old rats (Rangaraju et al., 2009), the steady-state levels of phosphorylated HSF1 (Figure 4C, arrow) are elevated in the nerves of 1-year-old C22 mice. Target genes of HSF1, including HSP104, HSP90 and HSP70, and HSP40 also appear higher at all studied ages in samples from affected mice as compared with Wt. This is consistent with our previous reports on nerve samples from 3-month-old C22 mice (Amici et al., 2007) and isolated C22 Schwann cells (Rangaraju et al., 2008). The roles of small chaperones HSP27 and αB-crystallin (αBCryst) in Schwann cell biology are largely unknown, but mutations in HSP27 are associated with CMT4 and distal hereditary motor neuropathies (Rossor et al., 2012). While in whole nerve lysates the origin of these chaperones can be axonal or Schwann cell-derived, they both appear slightly elevated in neuropathic samples, at all three ages examined (Figure 4D).

Since small chaperones are implicated in axonal neuropathies (De Rijk et al., 2000; Evgrafov et al., 2004), we used biochemical markers to examine potential alterations in axonal proteins (Figure 4E). First, we assessed whether the levels of hypophosphorylated heavy neurofilaments (NF-H), as detected with the monoclonal antibody SMI32, were altered. Positive staining with this antibody has previously been associated with segmental demyelination in multiple sclerosis (Trapp et al., 1998) as well as hypomyelinated axons in peripheral nerves (Pereira et al., 2010). In neuropathic nerves, there is a pronounced progressive increase in hypophosphorylated NF-H with age (Figure 4E), suggesting axonal pathology. Therefore we also examined the presence of spectrin isoforms, as elevated levels of cleaved spectrin serve as an index of calpain activation and axonal injury (Buki et al., 1999). Fraction of cleaved spectrin relative to the full-length isoform is already increased in neuropathic nerves at 2-months and further ascends (0.48 to 1.45) with disease progression (Figure 4E). A recent study described the occurrence of growth-associated protein, GAP43 (Grasselli et al., 2011), within axonal swelling of demyelinated fiber tracks in multiple sclerosis (Schirmer et al., 2013). In agreement, we also find a gradual increase in the levels of GAP43 in nerve lysates from neuropathic mice (Figure 4E), likely reflecting ongoing demyelination. Together, these results reveal aberrant expression of protein chaperones within neuropathic nerves, which in part originate from Schwann cells, but with time lead to axonal changes. The detected increase in the studied markers of axonal pathology supports the notion that the ongoing demyelination elicits axonal damage in neuropathic nerves.

### Schwann cell hyperproliferation and immune cell infiltration in affected nerves

**Figure 5:**
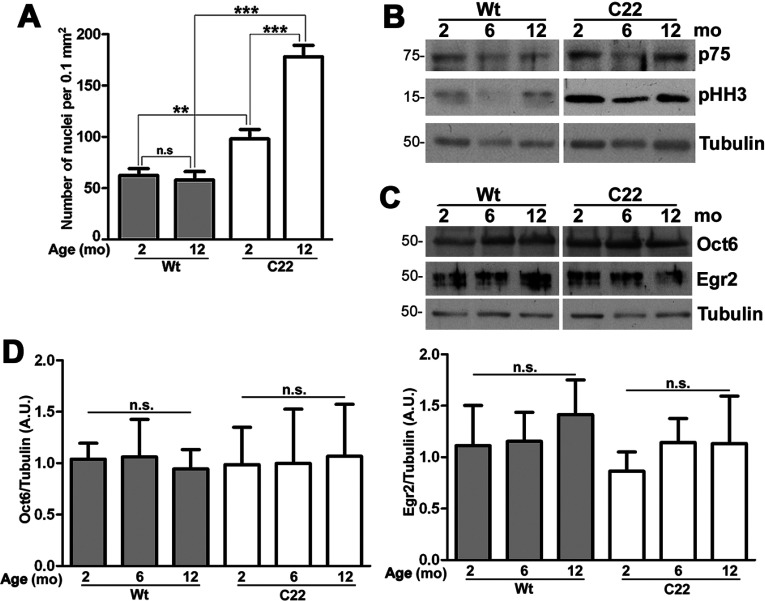
Schwann cell proliferation and dedifferentiation in nerves of neuropathic mice (**A**) Quantification of the number of nuclei in sciatic nerve sections (0.1mm^2^, per field area) from 2- and 12-month-old Wt and C22 mice (*n*=3–4 per group). (**B**) Whole sciatic nerve lysates (10 μg/lane) from Wt and C22 mice (*n*=3–4 per group) at the indicated ages were analyzed by Western blotting for p75 and phosphorylated histone H3 (pHH3). (**C**) The nerve lysates (15 μg/lane) were also probed for the transcription factors Oct6 and Egr2. Tubulin is shown as a protein loading control (**B** and **C**). Molecular mass is shown on the left, in kDa. (**D**) Semi-quantitative analyses of Oct6 and Egr2 levels after correction for tubulin from four independent experiments are shown. Unpaired Student's *t* test; **P<0.01; ***P<0.001; n.s., not significant; mean±S.E.M.; AU, arbitrary units.

Axo-glial signaling is an essential component during myelination and in maintenance of intact myelin (Jessen and Mirsky, 2005). In response to axonal injury or damage, Schwann cells de-differentiate and proliferate, followed by remyelination of regenerated nerves (Chen et al., 2007). To examine the differentiation state of Schwann cells concomitant with the axonal pathology (Figure 4E), we labeled nerve sections with Hoechst dye and counted the number of nuclei, excluding epi- and endo-neurial cells (Figure 5A). The average number of nuclei within fixed nerve tissue areas of 2-month-old Wt mice is 62.4±6.8 and is maintained at 58.0±8.2 at 12-months. In nerves from C22 mice, there are higher numbers of nuclei even at 2-months (98.0±9.3), which is statistically significant compared with age-matched Wt. Furthermore, there is a nearly 2-fold increase in the number of Schwann cell nuclei in the oldest C22 samples (178.0±11.2), as compared with 2-months and this increase is also significant when compared with 12-month-old Wt. We also examined biochemical markers of Schwann cell differentiation and proliferation using Western blots (Figure 5B). The Schwann cell marker p75, is nearly undetectable when the cells are myelinated (Jessen and Mirsky, 2005), just as seen in samples from Wt animals. In the C22 animals, higher levels of p75 are observed at all three ages compared with the Wt, likely as a reflection of ongoing demyelination. Correspondingly, the levels of phosphorylated histone H3 (pHH3), a mitotic marker, are low in nerves from Wt mice but are sustained at elevated levels in the neuropathic samples (Figure 5B). Next we examined the expression of transcription factors that regulate the myelinating Schwann cell phenotype, octamer-binding transcription factor 6 (Oct6) and early growth response 2 (Egr2/Krox20) (Jessen and Mirsky, 2005) (Figure 5C). The steady-state levels of Oct6 and Egr2 are sustained across the studied ages in samples from Wt mice, an observation that is confirmed by semi-quantitative analyses (Figures 5C and 5D). Furthermore, even though we found evidence for Schwann cell dedifferentiation in C22 neuropathic nerves, the levels of Oct6 and Egr2 are sustained during the studied time period (Figures 5C and 5D). This finding is consistent with reports from the CMT1A rat, where Schwann cell differentiation and myelination are uncoupled due to overexpression of PMP22 (Niemann et al., 2000).

**Figure 6:**
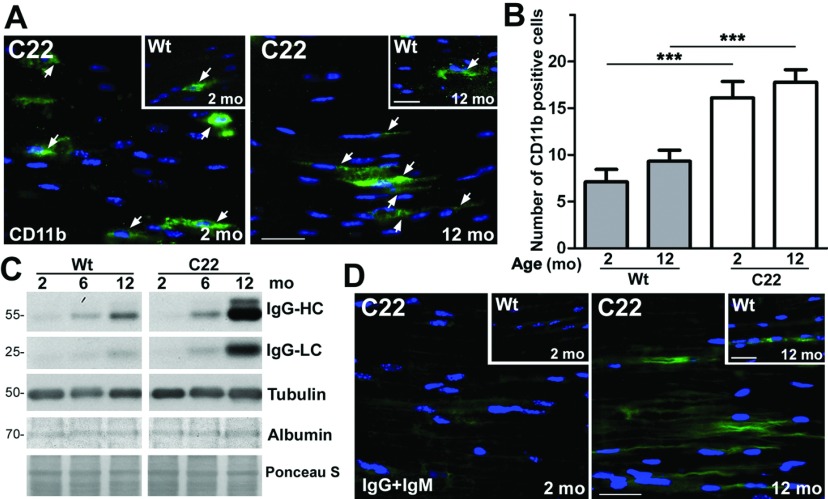
Macrophage infiltration of nerves in affected mice (**A**) Sciatic nerves from 2- and 12-month-old Wt (insets) and C22 mice (*n*=3 mice per group) were sectioned and stained for CD11b (green). Arrows mark CD11b-positive cells. (**B**) The number of CD11b-positive cells in sciatic nerves sections were counted in nine random fields (0.1 mm^2^, per field area) and graphed. Unpaired Student's *t* test; ***P<0.001; mean±S.E.M. (**C**) Whole nerve lysates (10 μg/lane) from 2-, 6- and 12-month-old Wt and C22 mice (*n*=3–4 per group) were analyzed for endogenous immunoglobulin G (IgG). The IgG heavy chain (IgG-HC) and light chain (IgG-LC) are shown. Tubulin is shown as a loading control. The levels of serum albumin were also analyzed in the same lysates. Ponceau S stained membrane is shown for protein loading. Molecular mass is shown on the left, in kDa. (**D**) Sections of sciatic nerves from 2- and 12-month-old Wt (insets) and C22 mice were reacted with antibodies against mouse immunoglobulins, IgG and IgM (green). Nuclei are labeled with Hoechst dye (blue) (**A** and **D**). Scale bar, 20 μm (**A** and **D**).

Upon axonal injury, Schwann cells attract immune cells through chemokines (Tofaris et al., 2002) to aid in the clearance of myelin debris (Martini et al., 2008). Previously, we observed elevated number of CD11b-positive marcophages in peripheral nerves of TrJ neuropathic mice (Misko et al., 2002), and of aged rats (Opalach et al., 2010). To assess macrophage infiltration into affected nerves, we immunostained sections with an antibody against CD11b (Figure 6A). While there are CD11b-postive immune cells within nerves of Wt mice, their frequency is noticeably higher in neuropathic samples (Figure 6A, arrows). Quantification of CD11b-positive cells in nine random fixed microscopic fields reveal a significant increase in C22 nerves, as compared with age-matched Wt (***P<0.001, Figure 6B). The pronounced increase in the number of nerve macrophages already at 2-months is in agreement with a previous report that counted F4/80-positive macrophages in femoral nerve sections of C22 mice (Kohl et al., 2010). In accord with the infiltration of immune cells, lysates from 2-, 6- and 12-month-old C22 animals contain elevated levels of mouse immunoglobulins (IgG), including IgG heavy (IgG-HC) and light (IgG-LC) chains (Figure 6C). While the levels of immunoglobulins increase with age in both genetic backgrounds, this trend is more pronounced in the neuropathic samples. To rule out potential contribution of increased vascularization of the nerve tissue, we stained the same samples with an antibody against serum albumin. As shown in the representative Western blot, the levels of albumin are comparable across the studied samples. Furthermore, the increase in endogenous Igs within neuropathic nerves is supported through immunohistochemistry, which detects increased Ig-like immunoreactivity with age in both genetic backgrounds (Figure 6D). In accordance with the biochemical data in Figure 6(C), there is a more noticeable increase in IgG- and IgM-like reactivity in the nerve samples from 12-month-old C22 mice, as compared with Wt. Together these results indicate that Schwann cell demyelinaton and de-differentiation in nerves of C22 mice are accompanied by immune cell infiltration, both of which are evident by 2 months of age during disease pathogenesis.

## DISCUSSION

Previous studies have examined behavioral, electrophysiological and morphological aspects of the neuropathy in PMP22-overexpressing C22 mice over a 1.5 year life-span and reported prominent axonal pathology (Verhamme et al., 2011). In the current study we focused on biochemical markers of protein quality control pathways, including chaperones and macroautophagy, which respond to the presence of overproduced, misfolded PMP22 within Schwann cells. While mis-trafficking of the overproduced PMP22 is evident early in the disease process, this pathogenic event leads to compromised proteasome activity and axonal injury only around 6 months of age, as detected by biochemical markers. Yet, throughout the examined life-span there is ongoing Schwann cell proliferation and immune cell infiltration. Together, these results indicate that the progression of neuropathy in C22 mice is underlined by multiple ongoing pathogenic mechanisms, including intrinsic subcellular events within Schwann cells and extrinsic soluble factor-mediated processes, such as macrophage infiltration.

*In vitro* overexpression and transgenic animal studies indicate that overproduction of PMP22 disrupts the trafficking of the protein to the plasma membrane, leading to myelin defects (Huxley et al., 1996; Fortun et al., 2006). In fact there is a clear dosage effect of the overexpressed PMP22, with higher copy numbers being more detrimental (Magyar et al., 1996; Huxley et al., 1998). While these transgenic rodent studies elegantly established the dosage-sensitivity of PMP22-linked diseases, it has been difficult to separate the contribution of the exogenous human and the endogenous mouse protein to the pathogenic events. To unravel this problem, we employed a number of unique anti-PMP22 antibodies that have preferential reactivity against the human or the rodent proteins. Using this approach, we found gradual accumulation of human PMP22 within affected mouse nerves, including a fast migrating incompletely processed ˜18 kDa form. In its mature ˜22 kDa form, PMP22 contains an N-linked complex carbohydrate modification and is reactive for the L2/HNK1 adhesion/recognition epitope (Snipes et al., 1993). How the misfolded, intracellularly retained PMP22 impacts Schwann cell protein homoeostasis and the myelination capacity of the cells is unclear, but likely involves inhibition of proteasome activity and entrapment of chaperones (Ryan et al., 2002; Fortun et al., 2006). Additional studies in samples from CMT1A patients will be required to ascertain if the subcellular pathogenic mechanisms seen in mice and cultured cells contribute to disease progression in humans.

Inhibition of proteasome activity appears to be a common mechanism by which aggregation-prone proteins impact disease progression. The proteasome is the primary defense mechanism in clearing misfolded, damaged peptides and is responsible for the turnover of short-lived proteins, including PMP22 (Pareek et al., 1997). Previously we have shown that in an *in vitro* cell culture model, expression of mutated PMP22 leads to a reduction in proteasome activity, as measured by degradation of a 26S ubiquitin-GFP reporter (Fortun et al., 2005). In our current study of C22 neuropathic nerves, we detected pronounced accumulation of ubiquitinated substrates at 6-months and a decrease in 20S chymotrypsin-like activity at 12-months. Age-related decline in proteasome activity has been observed in many different tissues (Conconi et al., 1996; Ponnappan et al., 1999; Keller et al., 2000), including in sciatic nerves of 29–38-month-old rats (Opalach et al., 2010); however, based on the results from Wt samples, aging was not the major factor during the studied life-span. Instead it is likely that accumulation of the aggregated PMP22 and other associated misfolded proteins contributed to this functional impairment.

Undegraded proteasome substrates are cleared from cells by the autophagy–lysosomal pathway (Nijholt et al., 2011). We have observed an analogous cross-talk between these two degradative mechanisms in the TrJ mouse model of PMP22 neuropathies (Fortun et al., 2003). In nerves of C22 mice, we similarly found elevated levels of LAMP1, CathD and Atg7, implying that in response to the misfolded, undegraded PMP22, Schwann cells synthesize more autophago–lysosomes to promote their removal. However, the accumulation of protein aggregates and the lower active/pro CathD and LC3 II/I ratios in neuropathic nerves with age suggests that this response does not go to completion. The stress of the overproduced, misfolded PMP22 might overburden the clearance mechanisms and impair their degradative capacity. In addition, similar to the proteasome, normal aging-associated impairment in the efficiency of the autophagy–lysosomal pathway has been observed in Schwann cells (Rangaraju et al., 2009), and we detected elevated levels of poly-ubiquitinated substrates in nerves of 12-month-old Wt mice. Therefore it appears that the combined effects of aging and neuropathic background lead to the accelerated accumulation of lipofuscin-like adducts in nerves of C22 mice.

Compared with the degradative mechanisms, it is more difficult to dissect the role of chaperones in the pathogenesis of PMP22-linked neuropathies. Previous studies have shown that chaperones co-localize with PMP22 aggregates in sciatic nerves of neuropathic mice, as well as in cell culture paradigms (Fortun et al., 2003, 2007). Similar to what has been observed in other protein misfolding disorders (Morimoto, 2011), the levels of HSP70 are elevated in the nerves of C22 mice, as compared with the age-matched Wt. A protective role for chaperones in neuropathies is supported by studies where exogenous induction of the heat-shock response either by diet restriction or pharmacologic modulation prevents the aggregation of PMP22 while promoting the myelination capacity of the Schwann cells (Rangaraju et al., 2008, 2009; Madorsky et al., 2009). In light of these results, why are the chaperones unable to prevent protein aggregation in neuropathic Schwann cells? Once again aging-associated changes in pathway activity, as well as potential alterations in substrate-recognition motifs within misfolded proteins might contribute. It is of note that, to date, calnexin is the only chaperone that has been shown to impact the subcellular trafficking of PMP22 (Jung et al., 2011), even though others are likely to be important.

Mutations in small chaperones have been associated with axonal neuropathies, possibly as a result of their interaction with the cytoskeletal elements (De Rijk et al., 2000; Evgrafov et al., 2004). We detected elevated levels of Hsp27 and αB-crystallin in affected nerves, but the cellular source for this response cannot be determined from the current biochemical studies. It is plausible that in response to demyelination there is a change in protein homoeostasis within the axons, which is supported by the pronounced axon pathology (Verhamme et al., 2011). Axonal damage is a well-accepted phenomenon in multiple sclerosis (MS) (Bitsch et al., 2000), a finding that supports commonality in disease mechanism between a CNS and PNS demyelinating disorder. In accordance, we also detected an increase in GAP43 expression in neuropathic nerves, which has been described as a marker of regenerative response in MS lesions (Schirmer et al., 2013). A third commonality between MS and demyelinating neuropathies is the inflammatory response, which appears to be an early event in the pathogenesis of the disease in C22 mice. In nerve injury and inherited demyelinating diseases, Schwann cells secrete cytokines and chemokines, such as M-CSF and MCP-1 (monocyte chemoattractant protein 1) to recruit immune cells to aid in the clearance of myelin debris (Martini et al., 2008; Kohl et al., 2010). While we detected CD11b-positive cells at 2-months of age in C22 mice, the increase in immunoglobulins became obvious only at later stages. This result is in agreement with the detection of macrophages prior to T-lymphocytes in mouse models of CMT1B (Schmid et al., 2000). While initially immune cells are recruited to clear myelin debris, studies in peripheral nerves of CMT1B and CMTX neuropathic mice show that lymphocytes and macrophages contribute to demyelination (Schmid et al., 2000; Maurer et al., 2002). This relationship is likely to hold true in C22 mice, as reducing MCP-1 was shown to ameliorate the demyelinating features in affected mice (Kohl et al., 2010).

Together our studies show that a number of ongoing pathogenic mechanisms contribute to the progression of the neuropathy in C22 mice, which initiates with abnormal expression of PMP22. While changes in protein homoeostasis elicit an early response in Schwann cells, axonal changes and immune cell infiltration quickly follow. The combined alterations in these various pathways seem to favor protein aggregation and demyelination, paving way for an auto-catalytic loop and thus contributing to disease advancement.
